# Surveying the Geneva impasse: Coercive care and human rights

**DOI:** 10.1016/j.ijlp.2019.03.001

**Published:** 2019

**Authors:** Wayne Martin, Sándor Gurbai

**Affiliations:** University of Essex, School of Philosophy and Art History, Essex Autonomy Project, Wivenhoe Park, Colchester CO4 3SQ, United Kingdom of Great Britain and Northern Ireland

**Keywords:** Human rights, mental health, consent, psychiatric coercion, psychiatric detention, Coercive treatment, United nations human rights committee, United nations convention on the rights of persons with disabilities, Non-consensual treatment, Involuntary treatment

## Abstract

The United Nations human rights system has in recent years been divided on the question as to whether coercive care interventions, including coercive psychiatric care, can ever be justified under UN human rights standards. Some within the UN human rights community hold that coercive care can comply with human rights standards, provided that the coercive intervention is a necessary and proportionate means to achieve certain approved aims, and that appropriate legal safeguards are in place. Others have held that coercive care is never justified. Disagreement over this issue has produced an impasse in the UN human rights system. We survey the impasse with particular attention to the legal arguments that inform the divergent positions. In doing so we introduce a distinction among a variety of different ‘abolitionist’ positions regarding coercive care, and draw a distinction between ‘non-consensual’ and ‘coercive’ treatment. We conclude with three proposals for moving beyond the current impasse.

## 1 Introduction

All over the world, governments and legislatures are considering whether and how to reform mental health and mental capacity legislation in order to ensure greater respect for human rights. In evaluating potential pathways for reform, it is natural to look to the UN human rights system for guidance. How should states parties design a human-rights-compliant framework for the provision and regulation of health care, including mental health care? Alas, what we find in the UN system is a notable lack of agreement among different treaty bodies and officials, particularly on the question of whether coercive care can ever comply with human rights standards. We refer to this situation as “the Geneva Impasse,” since the conflicting positions have been developed, articulated and adopted by UN Committees that meet in Geneva.^1^

Our aim in what follows is neither to settle this dispute nor to take sides in it. We propose instead to study the impasse with the tools of a surveyor, constructing a systematic mapping of the legal issues and arguments that have produced it. The initial task in this mapping exercise is to identify and specify the issue that has occasioned the impasse. What exactly do the different parties disagree about? In doing so we introduce a distinction among a variety of different “abolitionist” positions regarding coercive care, and draw a distinction between “non-consensual” and “coercive” treatment. We then go on to analyse the underlying legal structure of the impasse. Legally, the disputing parties share a common starting point: the rights enumerated in the several UN human rights covenants and conventions. So what is the legal reasoning that takes us from this common point of departure to conflicting conclusions? In addressing this question we clarify the notion of an “absolute ban” and its correlate, the concept of an “absolute right.”

Before turning to the matter at hand, three preliminary remarks may be in order. First, debates concerning coercive care sometimes operate with the implicit assumption that the issue arises only in the context of psychiatry. This is an assumption that we have taken care to avoid. Although there are some UN positions that are narrowly concerned with practices specific to psychiatric medicine, the positions that have produced the Geneva Impasse were for the most part carefully drafted so as to apply to any medical intervention – psychiatric or otherwise. In order to disrupt the entrenched assumption that the human rights issues in this area are confined to the context of mental illness, we shall make strategic use of two examples. One example comes from the context of psychiatric illness; the other has nothing to do with psychiatry.

Secondly, our analysis in what follows proceeds slowly, and we have tried to be as explicit as possible in defining key concepts and claims, and in providing fully explicit statements of key arguments. In places we review in detail matters that some readers may consider elementary; in other places we might be accused of belabouring the obvious. These have seemed to us to be risks worth taking. The matters with which we are concerned have occasioned considerable controversy; the impasse we are analysing is considered by many to be intractable; the relevant policy decisions could hardly be more important. We therefore seek the indulgence of readers in working through the issues and arguments one small step at a time.

Finally, our aim in this survey is not simply to document differences and disagreements, but also to look for points of actual or potential agreement. Our hope is that bringing the legal architecture of the impasse more fully into view might contribute towards the development of a common UN human rights position on a question of fundamental importance. We therefore conclude with three proposals for moving beyond the current impasse.

## 2 Mapping the impasse

In earlier work, we have reported in detail on a variety of different positions that have been taken by UN bodies and UN officials on the core question of coercive care interventions (Gurbai & Martin, 2018). For present purposes, we take our initial bearings from two formal UN documents adopted by two different treaty bodies within the UN human rights system.

The UN Human Rights Committee is the treaty body for the *International Covenant on Civil and Political Rights* (UN General Assembly, 1966; hereafter: ICCPR). In 2014, the Human Rights Committee adopted General Comment 35 (UN Human Rights Committee, 2014; hereafter: GC35), which provides guidance to states parties on compliance with ICCPR Art 9 (*Liberty and Security of Person*). This has direct relevance to our topic, insofar as coercive care practices intrinsically involve a deprivation of liberty. The final paragraph of GC35 bears directly on this issue:

The existence of a disability shall not in itself justify a deprivation of liberty but rather any deprivation of liberty must be necessary and proportionate, for the purpose of protecting the individual in question from serious harm or preventing injury to others. It must be applied only as a measure of last resort and for the shortest appropriate period of time, and must be accompanied by adequate procedural and substantive safeguards established by law (GC35, para 19).

This is a framework that we find reflected in existing legislation in many jurisdictions around the world – legislation that authorises coercive medical interventions when certain legal conditions are met and appropriate safeguards are in place.^2^

We can contrast this position with a statement adopted by the UN Committee on the Rights of Persons with Disabilities, which is the treaty body for the *UN Convention on the Rights of Persons with Disabilities* (UN General Assembly, 2006; hereafter: CRPD). In 2015, the CRPD Committee adopted a set of Guidelines concerning CRPD Art 14 (UN Committee on the Rights of Persons with Disabilities, 2015; hereafter: G14), which also concerns liberty. G14 includes the following passage:

The Committee has established that [CRPD] Article 14 does not permit any exceptions whereby persons may be detained on the grounds of their actual or perceived impairment. However, legislation of several States parties, including mental health laws, still provide instances in which persons may be detained on the grounds of their actual or perceived impairment, provided there are other reasons for their detention, including that they are deemed dangerous to themselves or others. This practice is incompatible with [CRPD] Article 14; it is discriminatory in nature and amounts to arbitrary deprivation of liberty (G14, para. 6).

Both of the quoted passages are addressed to the question of detention rather than care or treatment per se. But they already allow us to see the basic shape of the Geneva Impasse. The watershed question might be posed as follows: *Can coercive treatment ever comply with UN human rights standards?* The answer from one part of the UN human rights system seems to be: “Yes, provided that certain conditions are met.” But another part of the same system seems to be pointing towards an exceptionless “No.”

This ‘watershed question’ is one on which it is difficult to foresee compromise. As posed, our question is a clear example of what grammarians call a “closed question.” That is, the only possible answers are YES or NO. In considering one's answer, it is crucial to pause over the word “ever.” If we think that there are *any* circumstances where coercive care is justified, then our answer to the watershed question must be YES; the challenge is then to articulate a legal standard that defines and delimits those circumstances. To answer NO is to conclude that coercive care is *never* justified. That makes the impasse look intractable, and can make compromise between the two positions seem impossible.

## 3 Abolition: a buyer's guide

In trying to map the impasse more exactly, it will be worthwhile to consider how one might operationalise the negative answer to the watershed question. Following Wilson, 2018, we will refer to the negative answer as the “Abolitionist” position, since it calls for the abolition of a whole array of status quo practices, institutions and statutes that are involved in involuntary admission and coercive treatment.^3^ But it is vital to recognise that Abolition can take a variety of different forms. We survey three initial options here, although we don't mean to suggest that this list is exhaustive.

The most extreme example of abolition is what we shall call “A1 Abolition.” To adopt this approach would be to abolish all involuntary admission and coercive care and adopt in its place the principle that:

(A1) No hospital admission or medical intervention shall be undertaken without prior valid consent.

It is important to recognise that A1 Abolition is a *very* extreme policy. Our first example brings this into sharp relief. Suppose that you are a paramedic, arriving at the scene of an accident involving a wheelchair-user and a bus. A disabled accident victim is unconscious. Given the circumstances, it is clearly not possible to obtain valid consent for medical treatment. Under A1 Abolition you would have to refrain even from basic first aid, much less admission to hospital.

If A1 Abolition seems too extreme, then we need to look for alternatives. Consider A2 Abolition:

(A2) No hospital admission or medical intervention shall be undertaken in the face of valid refusal.

On its face, A2 Abolition might seem similar to A1 Abolition. But notice that it makes all the difference to our paramedic. The unconscious accident victim can't consent, but neither is he refusing. So A2 Abolition leaves room for a paramedic to do her job: stabilise, transport, admit if appropriate.

In our view, A2 has obvious advantages over A1, but it is probably not radical enough to satisfy the principal advocates of Abolition. That is because of its reliance on the notion of valid refusal. At least under current law, a refusal of treatment is valid only if undertaken by someone with competence or “mental capacity.” A2 thus leaves open the possibility of coercive treatment in cases where capacity is found to be absent. It would therefore allow for the retention of locked wards, which Abolitionists typically want to abolish. A third form of Abolition removes this restriction:

(A3) No hospital admission or medical intervention shall be undertaken in the face of any dissent, resistance or objection.

A3 differs from A2 in setting aside any assessment of the validity of dissent or refusal. If a patient objects to treatment then, under A3, their resistance should never be overcome. Unlike A2, A3 could provide the basis for the abolition of locked wards.^4^

We return below to consider whether there might be other viable variants on the abolitionist position. But already we can use this initial typology in order to refine our survey of the Geneva Impasse. We cited above the Guidelines from the CRPD Committee on CRPD Art 14. But it is important now to consider those Guidelines in conjunction with a passage from the same committee's General Comment 1 (UN Committee on the Rights of Persons with Disabilities, 2014; hereafter: GC1). Although GC1 is principally concerned with CRPD Art 12 (Equal Recognition before the Law), it includes two paragraphs that concern Articles 14 (Liberty and Security of the Person) and 25 (Right to the Highest Attainable Standard of Health). The Committee there states:

States parties have an obligation to require all health and medical professionals (including psychiatric professionals) to obtain the free and informed consent of persons with disabilities prior to any treatment. In conjunction with the right to legal capacity on an equal basis with others, States parties have an obligation not to permit substitute decision-makers to provide consent on behalf of persons with disabilities (para. 41).

Notice that the first sentence in this passage (as drafted) is an unequivocal endorsement of A1 Abolition. That is, it calls for states parties to disallow any medical treatment in the absence of free and informed consent, which must itself be provided in advance of any treatment. Notice also that the scope of the Committee's claim encompasses *all health and medical professionals*; there is no restriction to the specific context of psychiatric care. Applied to the circumstance of our unconscious disabled accident victim, it would mean that no treatment could be provided by the paramedic. The second sentence in the passage effectively doubles down on this position by cutting off a possible workaround, blocking a route whereby someone else might consent on the accident victim's behalf.

## 4 Non-consensual, coercive and involuntary treatment

Before going further, it will be worthwhile to pause briefly over a matter of terminology. Pūras has described the impasse as concerning the question of *non-consensual* treatment (*supra*, fn 1). In various other documents and analyses, one finds mention of “involuntary treatment,” “coercive treatment,” or “forced treatment.” These terms are sometimes used interchangeably, but we find it useful to draw a distinction among them.

At least as we shall use the term, ***non-consensual treatment*** is any treatment that is undertaken *in the absence of valid consent* (Non-Consensual = Without Consent.) But it is important to appreciate that not all non-consensual treatment is *coercive* or *forced*. Suppose for example that the paramedic in our example had proceeded to provide emergency medical treatment to the unconscious accident victim – contrary to the explicit requirements of GC1 as drafted and adopted. That treatment would be non-consensual, since no valid consent was obtained, but it does not seem right to describe it as coercive or forced. After all, the accident victim is not resisting, and (at least in the absence of contrary evidence) it is entirely reasonable for the paramedic to assume that the victim would indeed have consented to necessary and appropriate medical treatment had he been able to do so. So we need a way of distinguishing between the broader class of *non-consensual* treatment and the narrower subset of such treatment that is aptly described as *coercive*. Both the broader class and the narrower subset raise important human rights issues, but the issues are not always the same.

There are a variety of ways in which such a distinction might be drawn. For example, one might define care as coercive only when the patient is actually objecting, and treatment is imposed over those objections. Alternatively, one could adopt a broader definition, which would include not only actual objection but “counter-factual objection.” On this approach, care would count as coercive if the patient either actually objects or *would* have objected had he been able to do so.^5^ Our approach will be to define treatment as ***coercive*** (or *forced*) insofar as it is contrary to the reasonably ascertainable will or preference of the patient. Actual objection, under this definition, would be one form of evidence concerning the will and preference of the person, but treatment might be contrary to someone's reasonably ascertainable will or preference even though they are not objecting.

For the purposes of the present discussion, we have decided to avoid the term “involuntary treatment.” We are not convinced that there is a legally significant third category beyond the two that we have already defined. Moreover, in our discussions with stakeholders, we have found that “involuntary treatment” is used ambiguously – sometimes as equivalent to “non-consensual” and sometimes as equivalent to “coercive” (in the sense we have defined it). Because of this ambiguity we find it safest to avoid the term altogether. However, given our definition of “coercive” in terms of “contrary to the will,” and given the etymology of the term “involuntary” (which comes from the Latin, *voluntas* – the will), there is some reason to treat “involuntary treatment” as a synonym for “coercive treatment.” (Coercive/Involuntary = Contrary to Will or Preference.)

With these distinctions in hand we can now give greater precision to our mapping of the Geneva Impasse. The CRPD Committee has adopted an A1 Abolitionist position — forbidding both non-consensual and coercive treatment. The Human Rights Committee, by contrast, enumerates a set of conditions and safeguards under which not only non-consensual but also coercive treatment might be permissible.

## 5 Explaining the impasse: two approaches

The next task is to try to understand how we arrived at this impasse. What is the source of the schism?^6^ This is a question that we have posed in private conversations with a variety of stakeholders in the UN human rights community. The explanations that have been offered have for the most part been broadly sociological in nature. That is, the source of the disagreement is traced back to the social and professional positions of the persons involved in formulating the divergent Comments and Guidelines. Hence for example, we have been told that “the membership of the two Committees differs,” that “the CRPD Committee has high representation from Persons with Disabilities,” or that “the CRPD Committee lacks medical representation.” One former member of the Human Rights Committee has written that “specialized treaty bodies may lack legal expertise [or] pay insufficient attention to the rights of others that may come into conflict with the rights within their specialized mandate” (Neuman, 2018, 42).^7^

These sociological explanations are interesting and important, but we should not let them distract us from the underlying *legal* structure of the impasse. Our primary concern here is to identify the different paths of legal reasoning whereby the two Committees warrant their respective positions. In addition to the intrinsic importance of that legal reasoning, there are three reasons to adopt this approach. Firstly, the disputed question is ultimately a legal one: the issue is about what policies are lawful under a set of agreed international legal norms. Secondly, if we can get clear about the nub of the legal disagreement, this might itself suggest ways of breaking through the impasse. But there is also a third, and more troubling reason to adopt this approach. If indeed there are sound legal arguments in support of two contradictory positions on this policy question, this would indicate that the enumerated rights of the several UN treaties and conventions themselves form a logically inconsistent set. That would be a significant finding in its own right.

So what is the legal reasoning that lies behind the two conflicting conclusions? Alas, it is not entirely straightforward to find out. One lamentable feature of the relevant UN documents is that they are often quite thin on explicit legal argumentation. The two committees adopt statements of legal *conclusions* either without any argumentation at all, or with arguments that are at best enthymematic. In the face of this lacuna, we shall adopt an approach that we have used in other work (See for example: [Bibr bb0070][Bibr bb0065]). In the next three sections, we survey three different ways of understanding the legal argumentation that has produced the impasse. In doing so we are seeking to provide an explicit articulation of reasoning that is left largely implicit in the documents and positions adopted by the two UN bodies. Our reconstructions are guided by two closely connected desiderata: legal soundness and hermeneutic fit. That is, we are looking for the strongest possible legal arguments for the conclusions reached by the two Committees, and we are looking for reconstructions that help make sense of the various claims and statements that they have adopted.

## 6 A proportionality interpretation

A first way to understand the legal structure of the impasse would be to trace it back to two diverging applications of a proportionality exercise. Because UN human rights treaties and conventions enumerate a multiplicity of human rights, circumstances arise where rights conflict with one another. One familiar legal response to such circumstances is to engage in a proportionality exercise: compromises on some rights may be permitted if the policy adopted is a proportionate measure for protecting one or more fundamental rights (see for example: Klatt & Meister, 2012). Crucially, however, there is no algorithm for proportionality tests, and fully informed parties may reasonably disagree about cases.

In order to see how the proportionality framework might make sense of the Geneva Impasse, it will be useful to introduce our second example. Think about a person with bipolar disorder, entering an acute manic state. She knows (and the family knows and the care team knows) that her acute manias can lead to very severe harm to herself or to others; they can even threaten loss of life. Moreover, her ability to assess risks is profoundly impaired during her manic episodes. Yet she insists that she is not unwell, denies that she is entering a manic episode, and does not consent to treatment.

In considering whether to resort to coercive treatment in such a circumstance, we face a conflict of rights. How should we understand the conflict in this example? As a start, we can say that the person in question enjoys a right to life, and a right to the highest attainable standard of health. There may be circumstances where effective action to protect those rights in the context of a manic episode can only realistically be fulfilled through some kind of coercive intervention. Moreover, there may be circumstances where the rights of other people may be threatened unless a hospital admission or medical intervention is imposed. On the other hand, the person also has a right to liberty, a right to live in the community, a right to legal capacity on an equal basis with others, a right to respect for her physical and mental integrity. Coercive intervention would compromise those rights and potentially many others.

Our first reconstruction understands the divergence between the two UN positions as arising from divergent answers to this conundrum. What is the appropriate response to this conflict among rights? The Human Rights Committee holds that there are some circumstances where a suitably circumscribed limitation of rights to liberty, life in the community and legal capacity may be a *proportionate response* to a threat to life, health, etc. The CRPD Committee comes to the opposite conclusion: coercive care is a *disproportionate* measure for the protection of those rights. The debate becomes intractable insofar as there is an intrinsically subjective component to legal judgements of proportionality, and because the UN Human Rights system has no final authority to which appeal can be made in cases of divergent judgements.^8^

So how well does this first analysis satisfy our two desiderata? As regards the position of the Human Rights Committee, it does quite well. It provides a prima facie plausible legal argument in support of the position the Human Rights Committee has taken, and it also fits with the language used in articulating that position: notice in particular the prominence of the term “proportional” in the passage we have quoted from GC35. But the proportionality interpretation scores less well against our desiderata when it comes to the position of the CRPD Committee, and this for two reasons. First, we are not convinced that it provides the strongest possible legal argument for Abolition. And second, we are not convinced that it makes the best sense of the Guidelines on Article 14 adopted by the CRPD Committee. These two points will become clearer in the sections that follow, but the crucial point is that the proportionality interpretation leaves a gap in the legal argument for Abolition. This is because proportionality judgements are by their nature *particular*, and are therefore sensitive to the particular circumstances in any given case. In surveying the full range of cases, one will eventually come to circumstances where (for example) the imposition of a very small cost in liberty rights holds out the promise of a huge gain on right-to-life or protection-against-abuse. A stronger argument for an Abolitionist position would need to provide some reason to conclude, without giving consideration to the particulars of cases, that no such circumstance could ever warrant a coercive care intervention as a proportionate response to a threat to a fundamental right.

## 7 An absolute right interpretation

We turn now to a second construal of the legal divergence. The starting point is the same: a conflict among rights such as the one that arises in the circumstances of the manic episode. But the second interpretation then centres on the principle that not all conflicts of rights can be settled through a proportionality or “balancing” exercise. In particular, where an *absolute* right is at stake, no proportionality reasoning gets started. Hence if coercive care is torture, or tantamount to cruel, inhuman, or degrading treatment, for example, then it could *never* comply with human rights standards. Notice that this provides a different way of understanding the underlying legal structure of the Impasse. Our first mapping of the Impasse understood the two UN Committees as reaching divergent conclusions *within* the proportionality framework; this alternate mapping treats the Impasse instead as a dispute between the two Committees about *whether* the proportionality framework is legally appropriate to the question at issue.

We have used the rights concerning torture in elaborating this second legal schema, in part because it is the most familiar example of an absolute right, and in part because there are a number of academics and activists who have been making the argument that coercive psychiatry is indeed either torture or cruel, inhuman or degrading treatment (Minkowitz, 2007; Minkowitz, 2010; European Network of (Ex-) Users and Survivors of Psychiatry et al., 2012, paras. 17 and 28). Moreover, at least one judge in England has, in an obiter remark in a Court of Protection ruling, ‘judged it likely’ that forced feeding in the context of eating disorders can be tantamount to inhuman or degrading treatment.^9^

A great advantage of this second interpretation is that it provides a clearer path of legal reasoning in support of a strict Abolitionist position – precisely what seemed to be lacking in the logic of the proportionality interpretation. As in the case of slavery, an absolute right clearly warrants an exceptionless ban. So how does the second interpretation score against our desideratum of hermeneutic fit? As we have seen, we find clear evidence that the position adopted in GC35 reflects the conviction that a proportionality analysis is the appropriate legal framework for tackling these issues. So the hermeneutic fit with the statements from the Human Rights Committee is good. What about the CRPD Committee? Here the picture is mixed. In G14, the CRPD Committee enumerates specific practices (“seclusion and various methods of restraint in medical facilities, including physical, chemical and mechanic restrains [sic.]”) that it deems to be “not consistent with the prohibition of torture and other cruel, inhumane or degrading treatment or punishment against persons with disabilities” (para. 12). So it is clear that the CRPD Committee sees absolute rights as being relevant to the legal assessment of coercive care practices. But notice that this premise by itself could at most warrant an absolute ban *on the enumerated practices*; once again we are left with a gap in the argument for an absolute ban on coercion.

On this point, however, GC1 goes further. The CRPD Committee there provides a catalogue of the rights that are violated by “forced treatment by psychiatric and other medical professionals.” The catalogue includes “freedom from torture” (para. 42). If accepted, this claim would complete the argument for an absolute ban on coercion, and make a good fit with this second interpretation of the dispute. If *any* forced treatment is a violation of the right to be free from torture, then *all* forced treatment (whether “by psychiatric or other medical professionals”) must be banned. But this premise also marks the principal weakness of this second interpretation of the dispute. We can see this with reference to our example of the manic episode. In order to use this line of reasoning to warrant an absolute ban on coercive interventions in such a circumstance, the CRPD Committee would need to accept not only that *certain enumerated practices*, but that *any* coercive care intervention (no matter its nature, purpose, severity, duration, conditions, or manner of delivery) would be tantamount to torture or cruel, inhuman, or degrading treatment. This claim is fiercely disputed.^10^ A stronger legal argument would be one that relied on a less contentious premise.

## 8 An alternate absolute right interpretation

Our third interpretation echoes the second in locating the crux of the Impasse in a dispute about absolute rights and the applicability of judgments of proportionality. But instead of turning on the rights concerning torture or cruel, inhuman or degrading treatment (CRPD Art 15), the third interpretation turns on the question of whether there are absolute rights at work in CRPD Articles 12 (Equality before the Law) or 14 (Liberty and Security of Person). On this interpretation of the dispute, the Human Rights Committee is claiming that CRPD Art 12 and Art 14 do not express absolute rights; they can therefore be limited or restricted on the basis of a proportionality judgement. By contrast, the CRPD Committee is claiming that coercive care contravenes Art 12 or Art 14, that these articles express absolute rights, and that some form of Abolition is therefore the only option.

This third interpretation of the case for Abolition has advantages over the other two we have considered. Consider first the matter of legal soundness. Unlike the first interpretation, this interpretation avoids making the CRPD position rely on potentially irresolvable and implausibly a priori proportionality judgements regarding the human-rights-costs and human-rights-benefits of coercive care. It thus avoids the logical gap from which the first interpretation suffered. It has an advantage over the second argument because, in contrast to the rights concerning freedom from torture or cruel, inhuman or degrading treatment, it is self-evident that all coercive care implicates the core issues with which Articles 12 and 14 are concerned. After all, if care is undertaken contrary to the reasonably ascertainable will of the care-recipient, then it *ipso facto* constitutes a limit on the ability of the care-recipient to exercise legal capacity in the matter of their own care arrangements (Art 12); is also constitutes a very real limit to their liberty (Art 14).

What about the matter of hermeneutic fit? As with the first two interpretations of the dispute, the fit with the statements from the Human Rights Committee is good – for the same reasons that we have reviewed above. On the side of the CRPD Committee, the fit is tightest in connection with the Art 14 variant of the argument. In the passage from G14 that we cited at the outset, the key point to notice is the Committee's claim “that [CRPD] Article 14 *does not permit any exceptions*” (para 6). That looks like an attempt to treat CRPD Art 14 as expressive of an absolute right: no proportionality exercise should enter into policy making, and no threat to other rights can ever suffice to justify its curtailment. Notice furthermore that this claim comes in a section of the Guidelines that carries the heading: “The *absolute prohibition* of detention on the basis of disability” (emphasis added). Two paragraphs later, the same document refers to an “*absolute ban* of deprivation of liberty on the basis of impairment” (emphasis added). In a later General Comment we find a reference back to “the *absolute prohibition* of detention on the basis of disability as enshrined in Article 14” (UN Committee on the Rights of Persons with Disabilities, 2017, para 27, emphasis added). This language fits well with the third interpretation of the dispute, particularly as other absolute bans in human rights law (against slavery, against torture) are generally understood to be rooted in absolute rights. So the treatment of CRPD Art 14 as expressive of an absolute right makes good sense of the Committee's statements and the conceptual resources with which they are expressed.^11^

Of course the soundness of this line of legal argumentation must ultimately depend on establishing that CRPD Art 14 does indeed express an absolute right. This may seem implausible. The principal focus of Art 14 is the deprivation of liberty. It is universally agreed that that there are some circumstances in which a deprivation of liberty can accord with human rights standards. So it would be surprising, to say the least, to elevate CRPD Art 14 to the status of an absolute right! But on this point a crucial clarification is required. The foundational premise in this third interpretation of the CRPD Committee's argument for Abolition is *not* that there is an absolute right *not to be deprived of one's liberty*. The claim rather is that there is an absolute right not to be deprived of one's liberty *on the basis of a disability*. Otherwise put, it is only one specific clause of CRPD Art 14(1)(b) that is being treated as expressing an absolute right: “[T]he existence of a disability shall in no case justify a deprivation of liberty.”

## 9 Absolute rights in international law

In this section and the one that follows, we turn our attention directly to the concept of an absolute right. If our analysis so far is correct then the best interpretation of the specifically *legal* structure of the Geneva Impasse must focus as much on the *status* as on the *content* of CRPD Art 14. For the Human Rights Committee, Art 14 is one right (or *one cluster* of rights) among many. When Art 14 rights conflict with other rights the appropriate response is a proportionality exercise, which leaves open the possibility of a human-rights-compliant regime of coercive care. For the CRPD Committee, Art 14 articulates an absolute right that would rule out any proportionality justification *ab initio*. If indeed this is the best reconstruction of the dispute, then the Impasse directly implicates the concept of an absolute right.

We note first that the concept of an absolute right does not itself figure in any of the UN human rights covenants or conventions – nor indeed in other international human rights treaties. Since it is not used there, it is also not defined there. One might conclude on this basis that the concept is best avoided in the interpretation and application of UN human rights standards, and indeed some have argued that there are no absolute rights.^12^ But UN treaty bodies do regularly use the concept. For example, the UN Human Rights Committee denies that the right to life is an absolute right (UN Human Rights Committee, 2018, para. 10), while insisting that “the right to be tried by an independent and impartial tribunal is an absolute right that may suffer no exception.”^13^ The concept is also regularly used by the European Court of Human Rights (ECtHR), despite the absence of any explicit mention of absolute rights in the European Convention on Human Rights (ECHR).^14^

In the academic literature we find a range of definitions of the term. Shelton defines absolute rights by noting that “they apply without limitations clauses and without possibility of reservation, derogation, or denunciation, resulting in treaty-based ‘fundamental standards of humanity’” (Shelton, 2005, 18). Gewirth writes that “a right is *absolute* when it cannot be *overridden* in any circumstances, so that it can never be justifiably *infringed* and it must be *fulfilled* without any exceptions” (Gewirth, 1981, 2).

One thing to note about these definitions is that the concept of an absolute right is more stringent than that of a non-derogable right. Derogable rights are rights from which states are permitted to derogate, subject to certain conditions and constraints, in times of “public emergency which threatens the life of the nation” (ICCPR Art 4; see also Art 15 of the European Convention on Human Rights). Non-derogable rights are those which are not derogable in this sense; that is, they must be respected, protected and fulfilled even in times of public emergency. To describe a right as absolute is to go further: it is to say that no limitations or restrictions on the right would ever be justified.^15^ So while absolute rights are always non-derogable, non-derogable rights can be either absolute (e.g. freedom from torture or cruel, inhuman and degrading treatment or punishment) or non-absolute (e.g. right to freedom of religion).^16^

For our purposes it is not necessary to settle on any one definition of the term “absolute rights”; what matters are two key theorems that govern the use of the concept and that are consistent with all the published definitions:

T1 To every absolute ban there corresponds an absolute right (and vice versa).

T2 Where a right is absolute there is neither need nor scope for judgements of proportionality.

The point of T1 is that the concepts of an absolute ban (or absolute prohibition) and the concept of an absolute right are strict correlates. Hence for example if human beings have an absolute right not to be enslaved then slavery is absolutely prohibited (and vice versa). The point of T2 is that a right's status as absolute means that no proportionality reasoning can ever warrant its infringement. Hence for example we do not need to weigh up the potential human rights benefits of torturing a terrorism suspect in order to determine that doing so is prohibited under human rights standards.

## 10 Which rights are absolute?

We have argued that the underlying legal structure of the Geneva Impasse is best understood as a divergence between different UN bodies over which rights should be treated as absolute, i.e., as protected from any restriction or limitation on the basis of a proportionality judgement. As a final step in our survey it is therefore natural to consider the legal basis for a determination that an enumerated right in international law is or is not absolute. Alas, to the best of our knowledge, there is no agreed way of making such a determination. While there is a common list of “usual suspects” that appear in lists of absolute rights (torture; cruel, inhuman or degrading treatment; slavery; retrospective application of the criminal law …), little is said about how these lists are generated, or about how new candidates for a place on the honour roll should be vetted.

This in itself may be somewhat surprising. As we have seen above, UN treaty bodies regularly distinguish between absolute and non-absolute rights. And the distinction makes a difference! To treat a right as absolute is to grant it an exalted status in the hierarchy of rights; it is also to mark very rigid boundaries within which states parties must operate. So any system of human rights that relies on this concept would seem to require some method of adjudicating disputes about which rights merit this distinctive status.

Here again, alas, we find the lamentable practice that we encountered above: the statement of legal conclusions without articulation of the legal reasoning upon which those conclusions rely. A particularly telling example can be found in the case of *Gonzalez del Rio v Peru*, a case heard before the UN Human Rights Committee. The passage (cited above, *supra*, fn 13) in which an absolute right is asserted reads in full as follows: “The Committee *recalls* that the right to be tried by an independent and impartial tribunal is an absolute right that may suffer no exception” (emphasis added). But alas nothing in the judgement indicates from where this finding was ‘recalled,’ or on what basis it had originally been established.

For the sake of completeness, we note that the Vienna Convention on the Law of Treaties (UN Conference on the Law of Treaties, 1969; hereafter: VCLT) also says nothing about how to determine whether a particular right is absolute. The closest we find is a definition of a “peremptory norm of international law” – also known as *jus cogens*. The VCLT offers the following definition:

For the purposes of the present Convention, a peremptory norm of general international law is a norm accepted and recognized by the international community of States as a whole as a norm from which no derogation is permitted and which can be modified only by a subsequent norm of general international law having the same character (Article 53).

This is of limited use for our purposes. The first problem is that the standard invokes non-derogable rights, rather than the absolute rights that are contested at the Geneva Impasse. The second problem is that the VCLT standard for identifying *jus cogens* rights in effect requires recognition by the international community of States as a whole. This is not a standard that provides useful guidance in trying to resolve a substantive dispute in which authoritative representatives of the international community find themselves in disagreement!

In the face of this lacuna, we again adopt the approach of constructing arguments on either side of the question, guided by our two desiderata. In doing so, we can start by adopting a *via negativa*. For while there is no agreed method for determining that a particular right *is* absolute, there are clear ways of identifying rights that are *not* absolute. This can be done by looking to the particulars of the language in which rights are enunciated. In the UN human rights covenants and conventions, the specification of some rights includes explicit statements regarding restrictions, limitations or exceptions. (For one example, see *supra*, fn 16.) This clearly demonstrates that the underlying rights are not absolute. Other articles make strategic use of the words like “arbitrary,” which again signal that the underlying right is not absolute.^17^

Having established these negative criteria, one could then go further. Wherever we find the *absence* of these limiting formulations, this might be taken as prima facie evidence that the right in question is indeed to be understood as absolute. Finally, one could supplement this negative standard with a positive one. In this case one would be looking for formulations that signal exceptionless universality. For example: “*No one* shall be held in slavery. Slavery and the slave trade *in all their forms* shall be prohibited” (ICCPR Art 8.1). The strongest case in support of the claim that a particular right is absolute would then rest on a combination of (a) the absence of any of the usual signals of restrictions or limitations, and (b) the use of positive signals of exceptionless status.

How would CRPD Art 14 fare when assessed against this standard? The Article as a whole would clearly not pass this test, insofar as it requires states parties to ensure that persons with disability are not *unlawfully or arbitrarily* deprived of their liberty. These are canonical signals of a restricted or limited right. But recall that the strongest argument for the CRPD Committee's abolitionist position does not rest on the claim that Art 14 *as a whole* expresses an absolute right. The claim is rather that one clause *within Art 14* articulates such a right. When we look to that one clause (“[T]he existence of a disability shall *in no case* justify a deprivation of liberty”) we find that each of the two conditions are met. Negatively, we find no signal of the need to recognise limitations or restrictions. Positively we find a form of words (“in no case”) that signals exceptionless universality.

What about the other side of the argument? What evidence might be advanced in rebutting the assertion of absolute status? Some have argued that there are no absolute rights (*supra*, fn 12). As we have seen, the UN covenants and conventions make no use of this terminology, and provide no explicit standard for distinguishing absolute from non-absolute rights. This is in striking contrast to the case of non-derogable rights, where the concept does occur in treaties, along with an explicit distinction between the rights that have and lack this status. This might be taken as evidence that no rights are granted absolute status within the UN human rights system. Whatever one thinks of the strength of this argument, however, it clearly does not satisfy our desideratum regarding hermeneutic fit. For as we have seen, the Human Rights Committee explicitly relies on the assertion of absolute rights in other contexts (*supra*, fn 13).

An alternate rebuttal might accept that there are absolute rights, and indeed accept the test for absoluteness as outlined above, yet deny that any right in CRPD Art 14 meets that standard. The claim here might be that the clause under discussion appears within an Article that includes the canonical signals of limitation and restriction. A third alternative would be to accept that there are absolute rights while denying that any *superficial test* (i.e., a test that claims to “read off” absolute status from the decontextualized language of individual articles) can suffice to establish an absolute right. A proponent of this third rebuttal might go on to add that the closest explicit analogue to an absolute right in international human rights treaties is the *jus cogens* rights described in VCLT Art 53. This might be taken as grounds for concluding that something approximating an international consensus is the only way to validate a claim to absolute rights.

## 11 Three steps beyond the impasse

Having now completed our survey of the Impasse, it is time to consider what lessons it might teach us in looking for a way forward. It would of course be naive to hope that the legal analysis provided here could itself resolve what has become an entrenched and politically sensitive dispute both within the UN human rights system and more broadly in civil society. At most we have provided a guide to the underlying legal architecture of the controversy. But such a guide might prove useful in focusing discussion and debate on key areas where further analysis is required. We also believe it can help identify three significant points on which constructive dialogue might be expected to generate broad consensus. We certainly don't mean to suggest that these three steps could suffice to resolve all the differences, but we see the potential for them to form an agreed basis for continuing work towards a consensus UN position.

### 11.1 Step one: rule out A1 abolition

In looking to move beyond the current impasse, one significant obstacle concerns the position that we have described as A1 Abolition. As noted, this is a particularly extreme variant of the Abolitionist position, and would preclude the provision of even basic first aid to any person unable to give consent. In addition to the serious human rights violations that could be occasioned by such an approach, our own view is that it would be *unethical* to adopt such a policy. We also find it impossible to imagine the emergence of a democratic consensus supporting it. It is therefore encouraging to recognise that A1 Abolition is not actually entailed by *any* of the three legal arguments that we have reconstructed.

To see this, consider how each of the three legal arguments would bear upon our initial example of the paramedic and the unconscious disabled accident victim. As we have seen, A1 Abolition would prohibit any medical treatment in the absence of free and informed consent. But what would be the legal basis for such a prohibition? The provision of first aid to an unconscious accident victim cannot plausibly be described as a *disproportionate response* to the circumstance, which (*inter alia*) presents a real threat to the person's right to life and to the highest attainable standard of health. So the first argument does not justify a ban on a standard paramedic response. What about the second argument? In order to be applicable to this circumstance, we would have to claim that the paramedic's intervention amounts either to torture, or to cruel, inhuman or degrading treatment. But this is surely absurd. In fact, we submit, the paramedic's actions are an exemplar of *humane* treatment! What about the third argument, predicated on an absolute right not to be deprived of one's liberty on the basis of a disability? It also lacks application here, provided that the policies and practices of the paramedic services are applicable to all on an equal basis. So A1 Abolition simply cannot be warranted (much less mandated) on the basis of any of the arguments that we have reconstructed.

A1 Abolition is a position that continues to be espoused, whether explicitly or by implication, within the UN human rights system.^18^ But in light of what we have learned (both about its practical implications for standard emergency services and about the lack of any identifiable legal justification) it is worth considering the possibility that A1 Abolition is best understood as an inopportune statement of the CRPD Committee's intended position. In support of this admittedly bold suggestion we would add: (a) that the sentence advocating A1 Abolition was not included in the draft of GC1 that was originally made available to stakeholders for comment^19^; (b) that the final version of GC1 included a passage which seems to allow for medical interventions in certain cases where “it is not practicable to determine the will and preferences of an individual”^20^ – a policy which is prima facie inconsistent with A1 Abolition, which would disallow any intervention in the absence of prior valid consent; (c) that in its many Concluding Observations, the CRPD Committee has never called upon states parties to abolish standard emergency services that provide treatment to unconscious accident victims; and (d) that the CRPD Committee has already seen the need to correct an error that was introduced in the process of editing GC1.^21^ Perhaps the most hermeneutically charitable interpretation of all this evidence is that paragraph 41 of GC1 represents a drafting error, and that the CRPD Committee never intended to adopt A1 Abolition at all, nor to call upon states parties to adopt such an extreme policy.

In considering the merits of this suggestion, it is helpful to return to the distinction that we have proposed between non-consensual and coercive treatment (§4, above). As we have seen, the final language of GC1 calls for a ban on non-consensual treatment – i.e., on any treatment in the absence of prior free and informed consent. But elsewhere in GC1 we find traces of a narrower ban that would allow treatment in the absence of advance free and informed consent, provided that the treatment provided accords with the “best interpretation of will and preferences.”^22^ In the adopted version of GC1 there is an unresolved tension between these two policies. Indeed we would go further and argue that the two policies are inconsistent, as can be seen by considering what options each allows for the paramedic in our example. It is therefore worth considering whether the drafters of GC1 erred in those passages where they focused on non-consensual treatment when it was in fact only coercive treatment which they sought to disallow.

We therefore propose that a useful first step in advancing beyond the current impasse would be to initiate discussions that bring together both Abolitionists and representatives from the community of emergency first responders. Together, they might work through a range of real-world scenarios in order to consider what A1 Abolition would mean in practice for frontline emergency services. Support from human rights lawyers and activists would ensure that such a dialogue was informed by a complete survey of all possible arguments that might conceivably provide a legal basis for a policy of A1 Abolition. We anticipate that participants in such discussions will disagree about many matters of law and policy. But it is reasonable to expect that the discussion would generate a broad consensus that A1 Abolition is not the right solution to the policy challenges in this area, and is not required of states parties in order to achieve compliance with the CRPD.

### 11.2 Step two: focus on discrimination

If we could find a way past the obstacles associated with A1 Abolition, a second step beyond the impasse would be to identify potential alternatives. Here again our legal analysis provides a useful lead. What that analysis demonstrates is that the strongest Abolitionist argument with the best hermeneutic fit relies on a principle of non-discrimination in the deprivation of liberty. Notice, however, that none of the three Abolitionist positions that we distinguished at the outset reflect this core concern with non-discrimination. It therefore makes sense to introduce a fourth form of Abolition that reflects a tighter link between the proposed policy and the legal argument upon which it is predicated:

(A4) No hospital admission or medical intervention shall be undertaken on the basis of a policy that discriminates on the basis of a disability.

It is worth considering whether some variant of A4 Abolition might provide the basis for a consensus in moving beyond the current impasse.

In doing so, we can start by applying A4 Abolition to the circumstances faced by our paramedic. Let's assume that the paramedic treats the disabled person at the scene and then facilitates admission to hospital – all without obtaining free and informed consent. Would her actions be permissible under A4 Abolition? Notice that in order to answer this question we need to know more than simply the facts of the particular case. (Does the accident victim have a disability? Was he able to give consent? Did he resist or object? What medical treatment did he require? Did he have any kind of advance directive?) All that information may be relevant, to be sure, but to determine the compatibility with A4 Abolition we also need to know about the broader policies and procedures under which the paramedic is operating. In particular, we need to know whether the “rules of engagement” (so to speak) for this particular paramedic service do indeed avoid disability-based discrimination. In this particular example, the answer is plausibly “yes.” The paramedic is providing non-consensual treatment to an unconscious disabled accident victim. But she is doing so *because he is an unconscious accident victim*. The fact of the victim's *disability* is not a determinant of her decision to act without free and informed consent, and she would have done the same if (for example) she had been uninformed about his disability, or if he had not had a disability at all.

In our view it is a great advantage of A4 Abolition that it both leaves room in principle for the paramedic's non-consensual intervention, and also directs our attention to these questions about the underlying policies and procedures upon which intervention is predicated. A further advantage of A4 Abolition concerns the matter of hermeneutic fit. Recall that G14 emphatically rules out deprivation of liberty *on the basis of disability alone*, as well as deprivation of liberty *on the basis of disability plus other factors*. But this leaves open a third alternative: a policy based *on other factors alone*. So G14 seems to leave open, perhaps even to point towards, some variant of an A4 policy.

As a second concrete step in working through the Impasse, therefore, we see the need for a collaborative exploration of what it would mean to articulate A4 Abolition in the form of concrete policy. If indeed A1 Abolition is considered untenable and unwarranted, the challenge would be to frame candidate policies that meet two conditions: (a) they permit non-consensual treatment (i.e., treatment in the absence of prior free and informed consent) under at least some conditions, and (b) they avoid discrimination on the basis of disability.

Work on this policy challenge would need to be informed by research on a technical question of human rights law. Within the UN human rights system there had been a broad and longstanding consensus in support of what Wouter Vandenhole has described as “the widely-used pragmatic definition of discrimination.”^23^ Under the pragmatic definition, *differential treatment does not amount to discrimination, provided that the differential treatment can be justified on a reasonable and objective ground*. This principle can be found enunciated, inter alia, in: Human Rights Committee, GC18^24^; UN Committee on Economic, Social and Cultural Rights, GC20^25^; Committee on the Elimination of Racial Discrimination, General Recommendation XIV.^26^ It is noteworthy that these several treaty bodies do not restrict this approach to the context of indirect discrimination. Even differential treatment that is expressly based on a protected characteristic can in principle be justified. If it is justified then, under the pragmatic definition, *it is not discrimination*.

In considering how A4 Abolition might be realised in policy, we see the need for a dialogue within the UN human rights community as to whether (and to what extent) this pragmatic definition remains valid. It is noteworthy that the UN sources that explicitly endorse the pragmatic approach all predate the adoption of the CRPD in 2006. So does the CRPD leave the pragmatic definition intact or does it entail some kind of qualification or restriction? This is one of those seemingly fussy legal technicalities upon which quite a lot turns in practice. A4 Abolition calls for an end to all coercive and non-consensual care policies that discriminate on the basis of disability. We see this as a strong candidate for a further point of consensus in finding a way beyond the present impasse. But there are potentially two quite different routes that states parties might take in trying to satisfy this condition. One strategy would be to formulate so-called ‘disability-neutral’ policies – policies that draw no distinctions on the basis of disability and apply to all on an equal basis.^27^ In our example of the manic episode, coercive or non-consensual care interventions would be permitted only if the policies upon which they are predicated apply to all on an equal basis. But if the pragmatic definition remains valid, then *a policy need not be disability-neutral in order to avoid disability-based discrimination*.^28^ This would leave open the possibility that states parties might continue to draw distinctions on the basis of disability while adhering to A4 Abolition, provided that the differential treatment was justified on a reasonable and objective ground. In the circumstances of the manic episode, this would leave room in principle for coercive or non-consensual interventions that are predicated upon the person's particular medical condition. In order to give shape and direction to A4 Abolition, there is therefore a need to revisit the pragmatic definition of discrimination in light of the provisions of the CRPD. Does it still apply where differential treatment is based on disability? Is it applicable where the differential treatment involves deprivation of liberty? Ideally, such a reassessment should involve representatives from the various UN treaty bodies which have endorsed the pragmatic definition in the past.^29^

### 11.3 Step three: towards a general comment on CRPD art 15

These first two steps beyond the impasse do not yet engage the issues concerning torture or cruel, inhuman and degrading treatment – i.e., the CRPD Art. 15 rights. This is an area where the impasse may prove the most intractable, and where strongly held opinions may be most sharply divided; some have suggested to us that there is simply no prospect for reaching a consensus. Before resorting to this despairing conclusion, however, we see both a need and an opportunity to work through the specifically legal arguments more fully.

One important lesson from our legal survey takes the form of a distinction. As we have seen, some discussions of these issues take an *enumerative approach* – listing specific practices of psychiatric care that amount to violations of CRPD Art 15 when imposed without consent. Other analyses adopt what we might call a *blanket approach*, claiming that any forced treatment is *ipso facto* an Art 15 violation. These two approaches are logically and legally distinct. As we have noted, only the second approach would suffice to underwrite an absolute ban on all coercive care. The enumerative approach could at most warrant a ban on the enumerated practices, perhaps along with other practices that are relevantly similar.^30^

The most explicit statement of the blanket claim appears in GC1, para 42. We reported on this passage above, but it is perhaps now worth quoting it in full.

As has been stated by the Committee in several concluding observations, forced treatment by psychiatric and other health and medical professionals is a violation of the right to equal recognition before the law and an infringement of the rights to personal integrity (art. 17); freedom from torture (art. 15); and freedom from violence, exploitation and abuse (art. 16).

It is important to notice that this claim exhibits two forms of generality. Note first that the claim is wholly lacking in any qualification or restriction. The claim is not that certain *particular clinical practices* violate CRPD Art 15, nor that forced *psychiatric* treatment is a violation of CRPD Art 15, nor that forced treatment is an Art 15 violation when imposed *on a discriminatory basis*. The claim is that *any forced treatment* is a violation of CRPD Art 15. The claim is also general in a second sense. It refers to “freedom from torture” and it cites CRPD Art 15, but it does not draw any distinction among the four rights that are articulated in CRPD Art 15: the right not to be tortured, the right not to be subjected to cruel treatment; the right not to be subjected to inhuman treatment; the right not to be subjected to degrading treatment. It therefore might be read as claiming that any forced treatment is tantamount to torture, or it might be read as claiming that any forced treatment is a violation of at least one of the four Art 15 rights – without specifying which one.

As a third step beyond the impasse, we see the need to achieve greater clarity about this difficult and contested issue by making use of the discipline of explicit legal argumentation. If the enumerative approach is taken, then the challenge is to be explicit about the rationale that informs the list of prohibited practices. This need not require the articulation of some rigid criterion or decision-procedure; that may well be unavailable in this case. But the work of making the rationale more explicit would help avoid the appearance of arbitrariness; seeking a reflective equilibrium between general principles and particular practices would facilitate extension of the enumerative approach to new cases. If a blanket approach is taken, then the aim should be to provide a full statement of the legal argument that warrants the blanket claim.

Work towards these goals can be greatly aided by drawing on other UN sources. CRPD Art. 15 does not include any definition of torture; neither does ICCPR Art 7. But a definition is provided in Article 1 of the *UN Convention against Torture and Other Cruel, Inhuman or Degrading Treatment or Punishment* (CAT).^31^ A number of UN reports have undertaken analysis of that definition and struggled with the challenge of applying it to cases.^32^ A particularly comprehensive analysis has been provided by the UN Voluntary Fund for Victims of Torture.^33^ The Human Rights Committee has on several occasions provided guidance on ICCPR Art 7, both in its General Comments 7 and 20 and in its rulings on specific complaints.^34^ A fully explicit legal argument on these issues would presumptively begin from CAT Art 1, engage with existing UN sources, and then undertake to demonstrate both that and why either some or all forms of coercive care do indeed fall under one or another of the categories of action that are banned under CRPD Art 15. This could form part of the preparatory work for an eventual General Comment on CRPD Art 15.

This is not the place to embark on that fine-grained analysis, but one final observation is perhaps worth adding. The definition in CAT Art 1 directs attention to four elements that must be taken into account in determining whether a particular act is tantamount to torture. The four elements are: (i) the nature of the act; (ii) the intention of the perpetrator; (iii) the purpose; and (iv) the involvement of public officials (*supra*, fn 31). The Human Rights Committee has provided a similar list, distinguishing (i) the nature, (ii) the purpose and (iii) the severity of the treatment applied (UN Human Rights Committee, 1992, para 4). These schemata provide a legal and logical structure for investigating and adjudicating allegations of violations of CRPD Art 15. Reliance on that structure may or may not lead to a consensus on the disputed question, but at the very least it could provide an agreed basis for gaining greater clarity where differences remain.

## 12 Conclusion

As we noted at the outset, it was Dainius Pūras who described the current situation in Geneva as an impasse. Pūras's, 2017 report concluded with the following call:

The Special Rapporteur seeks to develop, through an inclusive and participatory process and open dialogue, guidelines on human rights and mental health to support all stakeholders in the implementation of rights-based mental health policies in their respective areas of work. He welcomes contributions and suggestions in this respect. (*supra*, fn 1, para 90).

One of our aims in the foregoing survey has been to provide a set of analytical tools that might prove useful in the ongoing dialogue for which Pūras has called. We have proposed a distinction between non-consensual and coercive care. We have distinguished four distinct variants of the Abolitionist position. We have provided three reconstructions of the legal arguments upon which the divergent positions rely, and in so doing we clarified the concept of an absolute right and distinguished enumerative from blanket approaches in the application of CRPD Art 15. We have undertaken to disrupt the common misapprehension that the issues at stake at the Impasse pertain narrowly to coercive psychiatry. And we have outlined three concrete steps where we believe there is a real prospect for generating a broad consensus moving forward.

Our hope is that these contributions will be useful to those within the UN human rights system who are seeking a way out of the current stalemate. But our findings also have relevance beyond the ongoing discussions and debates in Geneva. The fundamental issues which have occasioned the Impasse are by no means exclusive to the UN context. They arise in debates in legislatures, government agencies and civil society organisations all over the world, as part of the ongoing, worldwide effort to develop new care practices that are fully respectful of human rights. In this effort, States parties are not in the end bound by the findings of UN treaty bodies. But they are bound by the human rights conventions to which they are party, and we share the view of those who hold that States parties have an obligation to engage seriously with the findings of treaty bodies with which they may disagree.^35^ Our survey of the Geneva Impasse is offered in support of those around the world who are struggling to fulfil that obligation.

Our contributions here are not meant to exhaust the space of possibilities. We are open to the possibility that there may be legal arguments that we have overlooked. And we recognise that there are further possible Abolitionist policies beyond those that we have surveyed here. We are under no illusions about the depths of continuing disagreement in this area of human rights law and practice. But we also see important opportunities for breaking through the current impasse by identifying significant points of agreement, gaining greater clarity about the legal issues and arguments, and embarking on specific steps which might reasonably be expected to resolve some existing disagreement and provide an agreed pathway for working on those that remain.

## Footnotes

1 In describing the current situation as an “impasse,” we follow Pūras: “At present, there is an impasse over how obligations in relation to non-consensual treatment are implemented in the light of the provisions of the Convention on the Rights of Persons with Disabilities, given the different interpretation by international human rights mechanisms.” Pūras, 2017, para 34.

2 Consider a representative example from the UK. Under the *Mental Health Act* for England and Wales, disability *alone* can never warrant a deprivation of liberty. But the presence of mental disorder in combination with other factors (notably ‘danger to self or others’) *can* be used to warrant detention and/or coercive care, provided that suitable legal procedures are followed and the prescribed safeguards are in place.

3 For an influential statement of the case for abolition, see Minkowitz, 2010.

4 A variant of A3 Abolition is defended in Brosnan & Flynn, 2017. See in particular page 66: “If there is any indication that the person is refusing treatment, this must be respected.” It is worth noting that respect in this context certainly would not preclude engaging in respectful and non-coercive persuasion and support.

5 In England and Wales, the 2008 edition of the Code of Practice for the Mental Health Act adopted a counterfactual approach in defining ‘objection.’ “[W]hether a patient is objecting has to be considered in the round, taking into account all the circumstances, so far as they are reasonably ascertainable. … If there is reason to think that a patient would object, if able to do so, then the patient should be taken to be objecting.” Department of Health, 2008, para 4.19. The most recent revision to the Code of Practice has removed this counterfactual test. Department of Health, 2015.

6 Open disagreement between treaty bodies within the UN human rights system has been remarkably rare. The case we are concerned with here is one of only a handful examples of which we are aware. The three other cases have concerned: (1) the permissibility of abortion in cases of foetal impairment, (2) the right to vote and to hold office, and (3) the permissibility of restrictions on legal capacity through the imposition of guardianship regimes. Concerning (1), the Committee on the Elimination of Discrimination Against Women, the Committee on the Rights of the Child, and the Committee on Economic, Social and Cultural Rights have called for access to abortion in case of foetal impairment (UN Committee on the Elimination of Discrimination against Women, 2013, para. 37(c); UN Committee on the Rights of the Child, 2011, para. 64(c); UN Committee on Economic, Social and Cultural Rights, 2009a, para. 25). By contrast, the Committee on the Rights of Persons with Disabilities has called for the abolition of any distinction in the period allowed under law within which a pregnancy can be terminated based solely on disability (UN Committee on the Rights of Persons with Disabilities, 2013a, paras. 14–15.). As regards (2), the UN Human Rights Committee has held that “established mental incapacity may be a ground for denying a person the right to vote or to hold office” (UN Human Rights Committee, 1996, para 4). However, the CRPD Committee stated that “a person's decision-making ability cannot be a justification for any exclusion of persons with disabilities from exercising their political rights, including the right to vote, the right to stand for election and the right to serve as a member of a jury” (GC1, para 48). Regarding (3), the Human Rights Committee's Concluding Observations have seemed to recognise the permissibility of guardianship arrangements (UN Human Rights Committee, 2009, para 19(a)), whereas GC1 rejects them.

7 Neuman was a member of the Human Rights Committee at the time when GC35 was drafted and adopted. In the same passage he goes on to write: “Of course, the HRC may also have misperceived empirical realities that the other treaty body understands better, or may have neglected the perspective of a disadvantaged group whose situation prompted the creation of the other treaty.”

8 The independence of the several human rights treaty bodies and their members is a declared principle of the functioning of the UN human rights system. See for example: UN General Assembly, 2014, paras 35–36, and UN General Assembly, 2012, Annex I, paras 2–7. Concerning the non-hierarchical structure of the UN treaty body system see for example, UN Secretariat, 2006, para 23. “The treaty body system has developed ad hoc and it does not function as an integrated and indivisible framework for human rights protection. […] A lack of coordination and collaboration among the treaty bodies may result in conflicting jurisprudence.”

9 *A NHS foundation trust v. Ms X* [2014] EWCOP 35: “repeated forcible feeding over a long period of time against her clearly expressed wishes, most especially with the use of physical restraint, is likely in my judgment to amount to inhuman or degrading treatment” (para 46).

10 As one indicator of just how contentious this premise is, notice that the UN Subcommittee on the Prevention of Torture not only rejects it, but also holds that there are circumstances where the *withholding* of non-consensual treatment could itself amount to a form of cruel, inhuman or degrading treatment or punishment. UN Subcommittee on Prevention of Torture and Other Cruel, Inhuman or Degrading Treatment or Punishment, 2016, para 15. See also below, §11.3.

11 We have not found analogous evidence to support the suggestion that the CRPD Committee treats Art 12 as expressive of an absolute right. In this context it is telling that while G14 uses the term “absolute” on three separate occasions, GC1 uses the term only once (GC1, para. 34) and then only in connection with the state's obligation to provide support for the exercise of legal capacity. For this reason we will focus in what follows only on the Art 14 variant of this third legal argument. However our analysis of the Art 14 variant applies, *mutatis mutandis*, to an analogous argument anchored in the assertion of an absolute Art 12 right.

12 See for example, Levinson, 1982; and Barak, 2012, 135.

13 *Gonzalez del Rio v Peru* (Views of 28 October 1992, Communication No. 263/1987, CCPR/C/46/D/263/1987), para 5.1.

14 The term “absolute right” has mainly been used by the ECtHR in connection with ECHR Art 3 (Prohibition of Torture), for example in *Gäfgen v. Germany* at para 176, and in *Saadi v. Italy* at para 127. In some cases other rights or certain elements of other rights are also claimed to be absolute. For example in *Ilaşcu and Others v. Moldova and Russia* (para. 334) the ECtHR treats the right not to be sentenced to death as absolute element of ECHR Art 2 (Right to Life). In a number of cases the European Court of Human Rights uses the term “absolute rights” in order to deny that a certain right is absolute, e.g. in the cases of *John Murray v. The United Kingdom* at para 47.

15 According to the UN Human Rights Committee, 2001, para. 7: “Conceptually, the qualification of a Covenant provision as a non-derogable … does not mean that no limitations or restrictions would ever be justified.”

16 ICCPR Art 4 explicitly includes ICCPR Art 18 among the non-derogable rights. But ICCPR Art 18 itself, which concerns freedom of thought, conscience and religion, expressly provides for the possibility of restrictions on those rights when “necessary to protect national security, public order, public health or morals, or the rights and freedoms of others” (ICCPR Art 18.3). Freedom of thought, conscience and religion are therefore non-derogable but non-absolute under the ICCPR.

17 ICCPR Art 6.1, for example, states that “No one shall be *arbitrarily* deprived of his life” (emphasis added). This formulation signals that the right to life is not absolute.

18 In May, 2018, the Office of the UN High Commissioner for Human Rights hosted a consultation on the theme: *Human Rights and Mental Health*. The recommendations that emerged from the consultation included the following: “States should ensure that *all* health care and services, including all mental health care and services, are based on the free and informed consent of the individuals concerned” (emphasis added). (UN High Commissioner for Human Rights, 2018, para. 46.) A year earlier, a report on the same topic from the High Commissioner recommended “an end to involuntary treatment.” In elaborating this recommendation, the report defined valid consent and involuntary treatment as follows: “In order for consent to be valid, it should be given voluntarily and on the basis of complete information on the nature, consequences, benefits and risks of the treatment, on any harm associated with it and on the availability of alternatives. Involuntary treatment refers to the administration of medical or therapeutic procedures without the consent of the individual.” The report goes on to state that CRPD Article 14 “precludes non-consensual commitment and treatment” (UN High Commissioner for Human Rights, 2017, paras. 17 and 29). Both reports therefore entail a commitment to A1 Abolition.

19 The history on this point is rather complicated. A draft of GC1 was adopted by the CRPD Committee at its tenth session (September 2–13, 2013), and an “Advance Unedited Version” was widely circulated for public comment. This version of the draft (UN Committee on the Rights of Persons with Disabilities, 2013b) seems no longer to be available on the UN website, but is available for download on the Essex Autonomy Project website (https://autonomy.essex.ac.uk/dgcart12auv). Notably, the words “prior to any treatment” do not appear in this draft. (See in particular, para. 37.) In the absence of these four words, the Draft General Comment did not include an unequivocal endorsement of A1 Abolition. A later version of the Draft General Comment was distributed on 25 November, 2013, and was included in the papers for the eleventh session of the CRPD Committee (30 March–11 April, 2014). This second version of the draft, which is available on the UN website (UN Committee on the Rights of Persons with Disabilities, 2013c), includes the words “prior to any treatment.” GC1 was formally adopted in April, 2014, although editing continued for some time thereafter.

20 GC1, para 21.

21 UN Committee on the Rights of Persons with Disabilities, 2018.

22 GC1, para 21; see also A/HRC/34/32, para 28.

23 Vandenhole, 2005, 71.

24 “Finally, the Committee observes that not every differentiation of treatment will constitute discrimination, if the criteria for such differentiation are reasonable and objective and if the aim is to achieve a purpose which is legitimate under the Covenant.” UN Human Rights Committee, 1989, para 13.

25 “Differential treatment based on prohibited grounds will be viewed as discriminatory unless the justification for differentiation is reasonable and objective. This will include an assessment as to whether the aim and effects of the measures or omissions are legitimate, compatible with the nature of the Covenant rights and solely for the purpose of promoting the general welfare in a democratic society. In addition, there must be a clear and reasonable relationship of proportionality between the aim sought to be realized and the measures or omissions and their effects. A failure to remove differential treatment on the basis of a lack of available resources is not an objective and reasonable justification unless every effort has been made to use all resources that are at the State party's disposition in an effort to address and eliminate the discrimination, as a matter of priority.” UN Committee on Economic, Social and Cultural Rights, 2009b, para 13.

26 “[A] differentiation of treatment will not constitute discrimination if the criteria for such differentiation, judged against the objectives and purposes of the Convention, are *legitimate* or fall within the scope of article 1, paragraph 4, of the Convention. *In considering the criteria* that may have been employed, the Committee will acknowledge that particular actions may have varied purposes. In seeking to determine whether an action has an effect contrary to the Convention, it will look to see whether that action has an *unjustifiable disparate impact* upon a group distinguished by race, colour, descent, or national or ethnic origin.” UN Committee on the Elimination of Racial Discrimination, 1993, para 2. (Emphasis added)

27 For three attempts to formulate disability-neutral policies that permit non-consensual interventions at least in some cases, see Gooding & Flynn, 2015, Flynn & Arstein-Kerslake, 2017, and Brosnan & Flynn, 2017. Gooding and Flynn propose the so-called ‘doctrine of necessity’ as a disability-neutral basis for interventions; Flynn and Arstein-Kerslake propose the criteria of ‘imminent and grave harm.’ Brosnan and Flynn would permit non-consensual interventions only in those life-threatening situations where communication cannot be established with the person, e.g. in the case of an unconscious person where paramedics' intervention is needed. However, they propose that in certain cases, including psychiatric treatment, non-consensual interventions should not be allowed at all. For a critical assessment of the doctrine of necessity as a disability-neutral approach, see Steele, 2017. For an analysis of Flynn & Arstein-Kerslake, 2017 and Brosnan & Flynn, 2017, see Minkowitz, 2017.

28 The general point can be illustrated with a stock example: a state might refuse to issue a driving licence to a blind person, or to someone subject to regular epileptic seizures, precisely on the basis of their blindness or epilepsy. This would not be a ‘disability-neutral’ policy, insofar as it singles out persons for differential treatment precisely on the basis of their disability, but it might nonetheless be a justifiable form of differential treatment. Under the longstanding principle, it would then not amount to discrimination. We are grateful to Lana Kerzner and Robert Dinerstein for suggesting these examples.

29 To date, the CRPD Committee has not adopted a formal position one way or another on the continued validity of the “pragmatic definition” of discrimination, nor on the general legal principle that follows from it. An early draft of its recent General Comment relied on a version of the pragmatic definition in the context of an analysis of indirect discrimination. https://www.ohchr.org/Documents/HRBodies/CRPD/GCArt5.docx. See para 20 (b). The version of the General Comment that was finally adopted did not include that passage, but neither did it expressly reject the pragmatic definition or its correlate principle. It is unclear whether this means that the Committee rejects the principle or whether it has so far failed to reach an internal consensus as to its validity in light of the CRPD.

30 For examples of the enumerative approach, see G14, para. 12, and Kooijams, 1986, para 119.

31 “For the purposes of this Convention, the term ‘torture’ means any act by which severe pain or suffering, whether physical or mental, is intentionally inflicted on a person for such purposes as obtaining from him or a third person information or a confession, punishing him for an act he or a third person has committed or is suspected of having committed, or intimidating or coercing him or a third person, or for any reason based on discrimination of any kind, when such pain or suffering is inflicted by or at the instigation of or with the consent or acquiescence of a public official or other person acting in an official capacity. It does not include pain or suffering arising only from, inherent in or incidental to lawful sanctions.” UN General Assembly, 1984, Art 1.

32 See for example Nowak, 2008.

33 UN Voluntary Fund for Victims of Torture, 2011.

34 UN Human Rights Committee, 1982; UN Human Rights Committee, 1992. (Note that GC7 was superseded by GC20.) For one example of a relevant ruling on a specific complaint, see UN Human Rights Committee, *Vuolanne v. Finland* (Communication No. 265/1987) CCPR/C/35/D/265/1987, 2 May 1989.

35 See Martin et al. (2016), Appendix A, and the literature cited therein.

## References

[bb0005] Barak A. (2012). Proportionality: constitutional rights and their limitations.

[bb0010] Brosnan L., Flynn E. (2017). Freedom to negotiate: A proposal extricating ‘capacity’ from ‘consent’. International Journal of Law in Context.

[bb0015] Department of Health (2008). Code of practice. mental health act 1983.

[bb0020] Department of Health (2015). Mental health act 1983: Code of practice.

[bb0025] European Network of (Ex-) (2012). Users and survivors of psychiatry, international disability alliance, mental disability advocacy center, and the world network of users and survivors of psychiatry. Submission to the UN Special Rapporteur on Torture on his Upcoming Thematic Paper on Torture in the Context of Healthcare.

[bb0030] Flynn E., Arstein-Kerslake A. (2017). State intervention in the lives of people with disabilities: The case for a disability-neutral framework. International Journal of Law in Context.

[bb0035] Gewirth A. (1981). Are there any absolute rights?. The Philosophical Quarterly.

[bb0040] Gooding P., Flynn E. (2015). Querying the call to introduce mental capacity testing to mental health law: Does the doctrine of necessity provide an alternative?. Laws.

[bb0045] Gurbai S., Martin W. (2018). Is involuntary placement and non-consensual treatment ever compliant with UN human rights standards? A survey of UN reports (2006–2017). Essex autonomy project.

[bb0050] Klatt M., Meister M. (2012). The constitutional structure of proportionality.

[bb0055] Kooijams P. (1986). Torture and other cruel, inhuman or degrading treatment or punishment. report by the special rapporteur appointed pursuant to commission on human rights resolution 1985/33. E/CN.4/1986/15.

[bb0060] Levinson J. (1982). Gewirth on absolute rights. The Philosophical Quarterly.

[bb0065] Martin W., Michalowski S., Jütten T., Burch M. (2014). Achieving CRPD compliance. Essex autonomy project.

[bb0070] Martin W., Michalowski S., Stavert J., Ward A., Ruck Keene A., Caughey C., McGregor R. (2016). Three jurisdictions report: towards compliance with CRPD art. 12 in capacity/incapacity legislation across the UK. Essex autonomy project.

[bb0075] Minkowitz T. (2007). The United Nations convention of the rights of persons with disabilities and the right to be free from nonconsensual psychiatric interventions. Syracuse Journal of International Law and Commerce.

[bb0080] Minkowitz T., McSherry B., Weller P. (2010). Abolishing mental health laws to comply with the convention on the rights of persons with disabilities. 2010: Rethinking rights-based mental health laws.

[bb0085] Minkowitz T. (2017). CRPD and transformative equality. International Journal of Law in Context.

[bb0090] Neuman L.G., Moeckli D., Keller H., Heri C. (2018). Giving meaning and effect to human rights: The contributions of human rights committee members. The human rights covenants at 50: their past, present, and future.

[bb0095] Nowak M. (2008). Interim report of the Special Rapporteur on torture and other cruel, inhuman or degrading treatment or punishment. A/63/175.

[bb0100] Pūras D. (2017). Report of the Special Rapporteur on the right of everyone to the enjoyment of the highest attainable standard of physical and mental health. A/HRC/35/21.

[bb0105] Shelton D. (2005). Are there differentiations among human rights? Jus cogens, core human rights, obligations erga omnes and non-derogability.

[bb0110] Steele L. (2017). Temporality, disability and institutional violence: Revisiting in re F. Griffith Law Review.

[bb0115] UN Committee on Economic, Social and Cultural Rights (2009). Concluding observations on the United Kingdom of Great Britain and Northern Ireland, the crown dependencies and the overseas dependent territories, E/C.12/GBR/CO/5.

[bb0120] UN Committee on Economic, Social and Cultural Rights (2009). General comment no. 20: non-discrimination in economic, social and cultural rights (art. 2, para. 2, of the international covenant on economic, social and cultural Rights), E/C.12/GC/20.

[bb0125] UN Committee on the Elimination of Discrimination against Women (2013). Concluding observations on the combined sixth and seventh periodic reports of the Dominican Republic, CEDAW/C/DOM/CO/6-7.

[bb0130] UN Committee on the Elimination of Racial Discrimination (1993). General recommendation XIV on article 1, paragraph 1, of the convention.

[bb0135] UN Committee on the Rights of Persons with Disabilities (2013). Concluding observations on the initial report of Austria, CRPD/C/AUT/CO/1.

[bb0140] UN Committee on the Rights of Persons with Disabilities (2013). Draft General comment on Article 12 of the Convention - Equal Recognition before the Law. Adopted by the Committee at its tenth session (2 – 13 September 2013). Advance unedited version.

[bb0145] UN Committee on the Rights of Persons with Disabilities (2013). General comment on article 12: Equal recognition before the law. Draft prepared by the Committee, CRPD/C/11/4. https://www.ohchr.org/Documents/HRBodies/CRPD/GC/DGCArticle12.doc.

[bb0150] UN Committee on the Rights of Persons with Disabilities (2014). General Comment No. 1 on Article 12: Equal recognition before the law (GC1), CRPD/C/GC/1.

[bb0155] UN Committee on the Rights of Persons with Disabilities (2015). Guidelines on Article 14 of the Convention on the Rights of Persons with Disabilities (G14).

[bb0160] UN Committee on the Rights of Persons with Disabilities (2017). General comment No. 5 (2017) on living independently and being included in the community, CRPD/C/GC/5.

[bb0165] UN Committee on the Rights of Persons with Disabilities (2018). General comment No. 1 (2014) Article 12: Equal recognition before the law. Corrigendum, CRPD/C/GC/1/Corr.1.

[bb0170] UN Committee on the Rights of the Child (2011). Concluding observations: Costa Rica, CRC/C/CRI/CO/4.

[bb0175] UN Conference on the Law of Treaties (1969). Vienna Convention on the Law of Treaties (VCLT), A/CONF.39/27.

[bb0180] UN General Assembly (1966). International covenant on civil and political rights (ICCPR), A/RES/21/2200.

[bb0185] UN General Assembly (1984). Convention against torture and other cruel, inhuman or degrading treatment or punishment (CAT), A/RES/39/46, annex.

[bb0190] UN General Assembly (2006). Convention on the rights of persons with disabilities (CRPD), A/RES/61/106.

[bb0195] UN General Assembly (2012). Guidelines on the independence and impartiality of members of the human rights treaty bodies (“the Addis Ababa guidelines”). Implementation of human rights instruments. Report of the Chairs of the human rights treaty bodies on their twenty-fourth meeting. A/67/222. Annex I.

[bb0200] UN General Assembly (2014). Resolution on strengthening and enhancing the effective functioning of the human rights treaty body system, A/RES/68/268.

[bb0205] UN High Commissioner for Human Rights (2017). Mental health and human rights, A/HRC/34/32.

[bb0210] UN High Commissioner for Human Rights (2018). Mental health and human rights, A/HRC/39/36.

[bb0215] UN Human Rights Committee (1982). General comment no. 7: Article 7 (prohibition of torture or cruel, inhuman or degrading treatment or punishment).

[bb0220] UN Human Rights Committee (1989). General comment no. 18: Non-discrimination.

[bb0225] UN Human Rights Committee (1992). General comment no. 20: Article 7 (prohibition of torture, or other cruel, inhuman or degrading treatment or punishment).

[bb0230] UN Human Rights Committee (1996). CCPR General Comment No. 25: Article 25 (Participation in Public Affairs and the Right to Vote), The Right to Participate in Public Affairs, Voting Rights and the Right of Equal Access to Public Service, CCPR/C/21/Rev.1/Add.7.

[bb0235] UN Human Rights Committee (2001). General comment no. 29: States of emergency (article 4), CCPR/C/21/Rev.1/Add.11.

[bb0240] UN Human Rights Committee (2009). Concluding observations of the Human Rights Committee on Russian Federation, CCPR/C/RUS/CO/6.

[bb0245] UN Human Rights Committee (2014). General comment no. 35, article 9 (liberty and security of person) (GC35). CCPR/C/GC/35.

[bb0250] UN Human Rights Committee (2018). General comment No. 36 (2018) on article 6 of the international covenant on civil and political Rights, on the right to life. CCPR/C/GC/36, Advance unedited version.

[bb0255] UN Secretariat (2006). Concept paper on the High Commissioner's proposal for a unified standing treaty body, HRI/MC/2006/2.

[bb0260] UN Subcommittee on Prevention of Torture and Other Cruel, Inhuman or Degrading Treatment or Punishment (2016). Approach of the subcommittee on prevention of torture and other cruel, inhuman or degrading treatment or punishment regarding the rights of persons institutionalized and treated medically without informed consent. CAT/OP/27/2.

[bb0265] UN Voluntary Fund for Victims of Torture (2011). Interpretation of torture in the light of the practice and jurisprudence of international bodies. https://www.ohchr.org/Documents/Issues/Torture/UNVFVT/Interpretation_torture_2011_EN.pdf.

[bb0270] Vandenhole W. (2005). Non-discrimination and equality in the view of the UN human rights treaty bodies.

[bb0275] Wilson K. (2018). The call for the abolition of mental health law: The challenges of suicide, accidental death and the equal enjoyment of the right to life. Human Rights Law Review.

